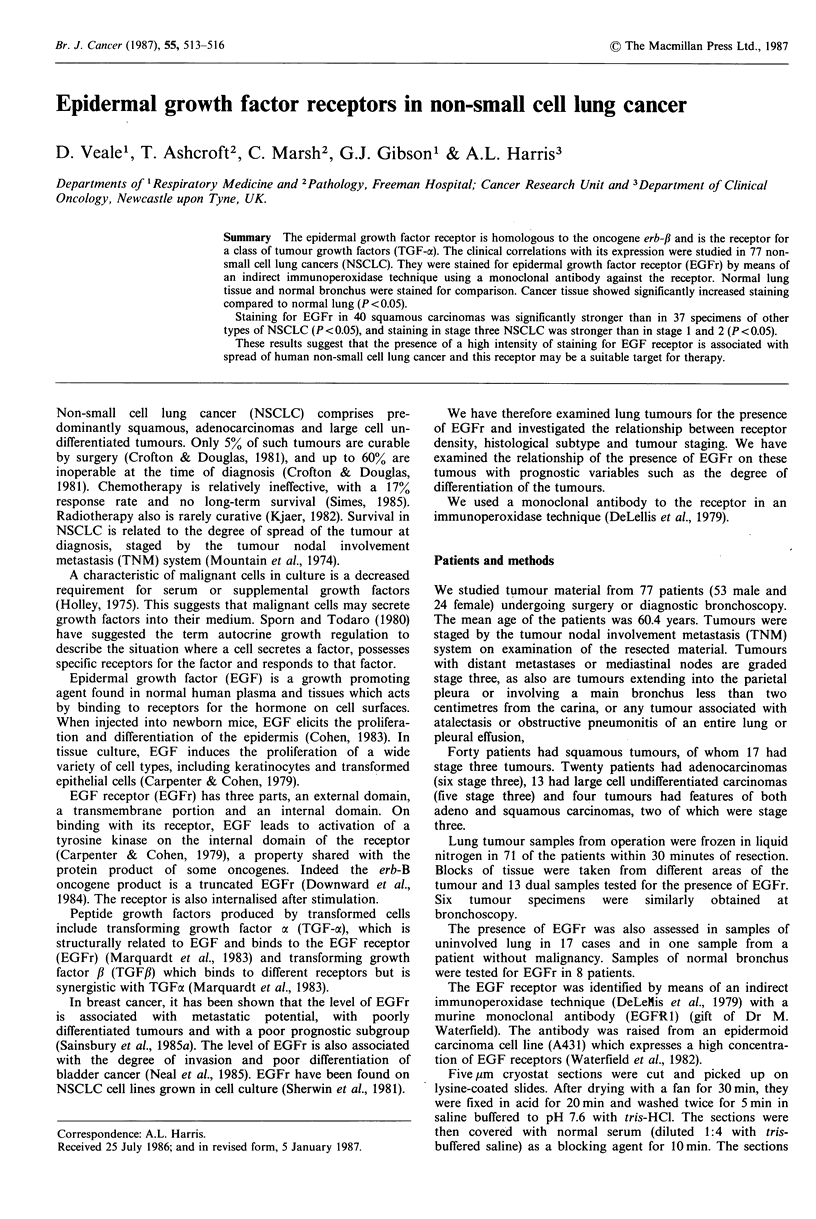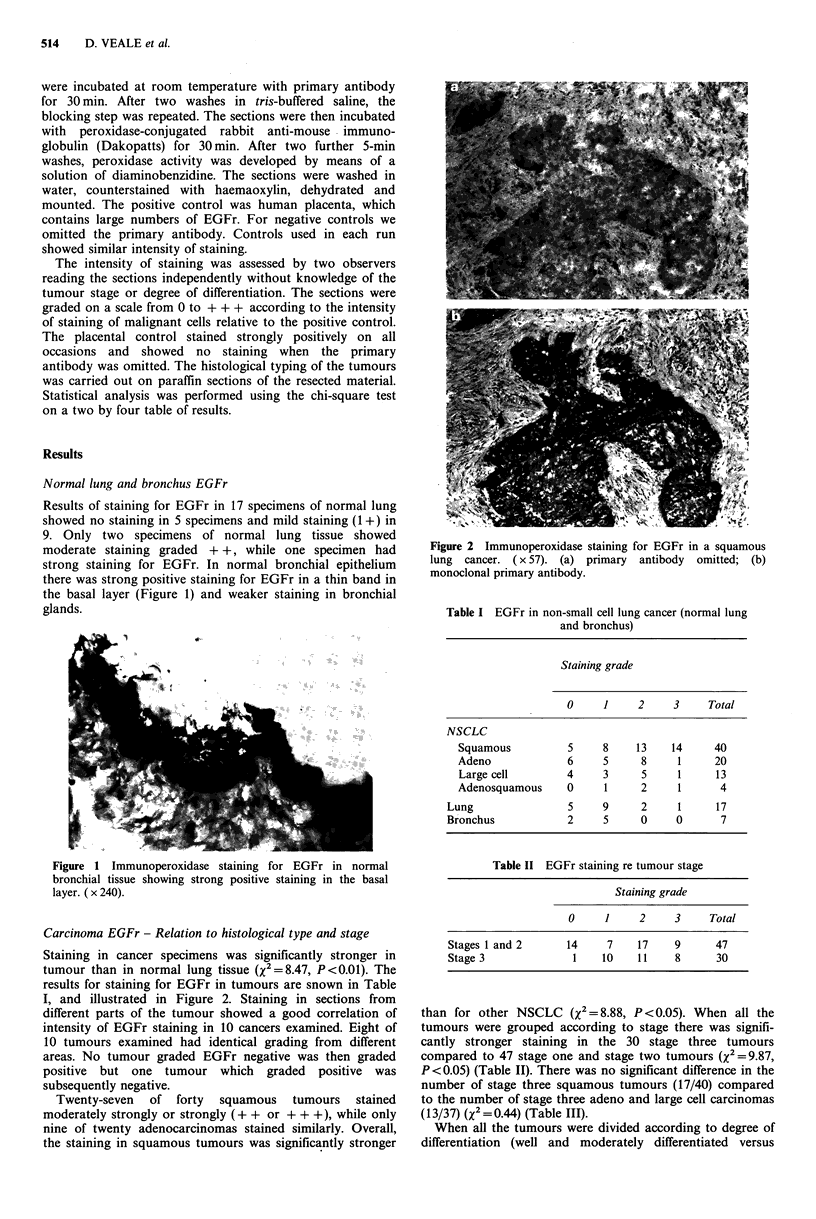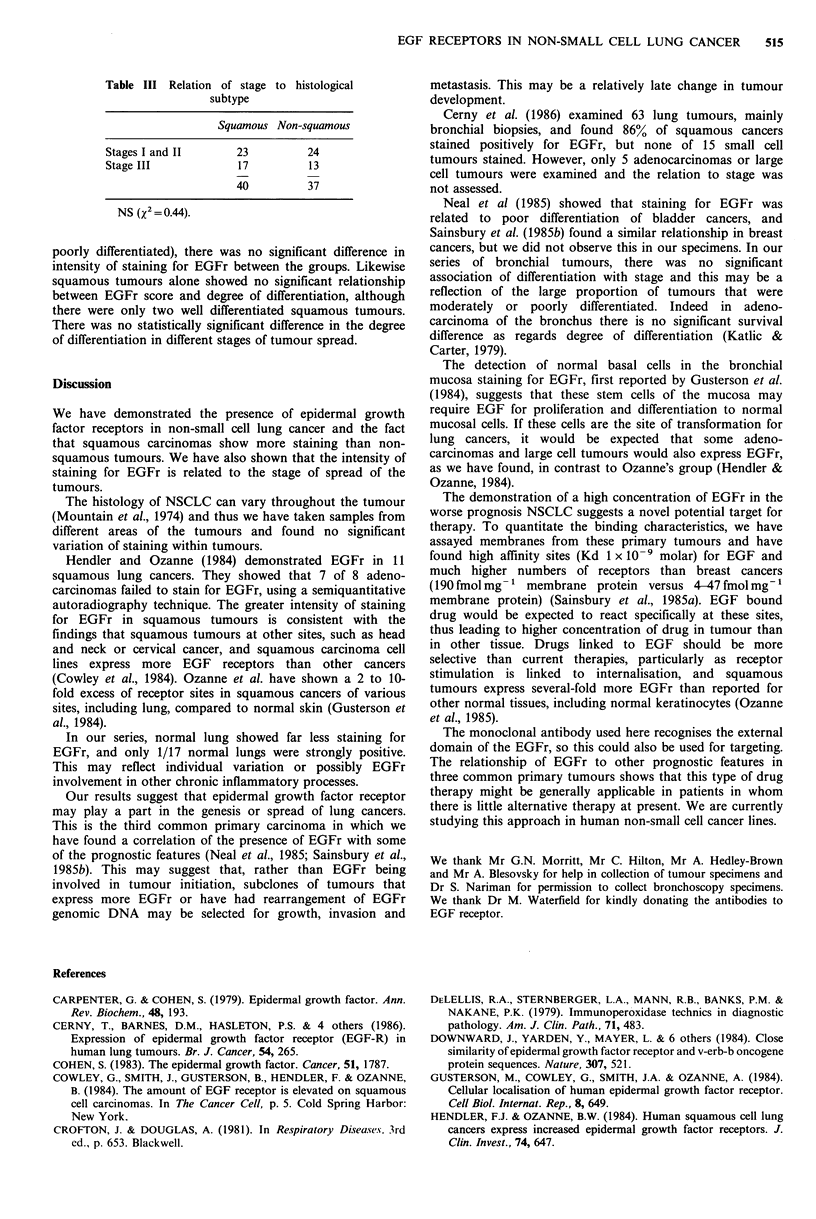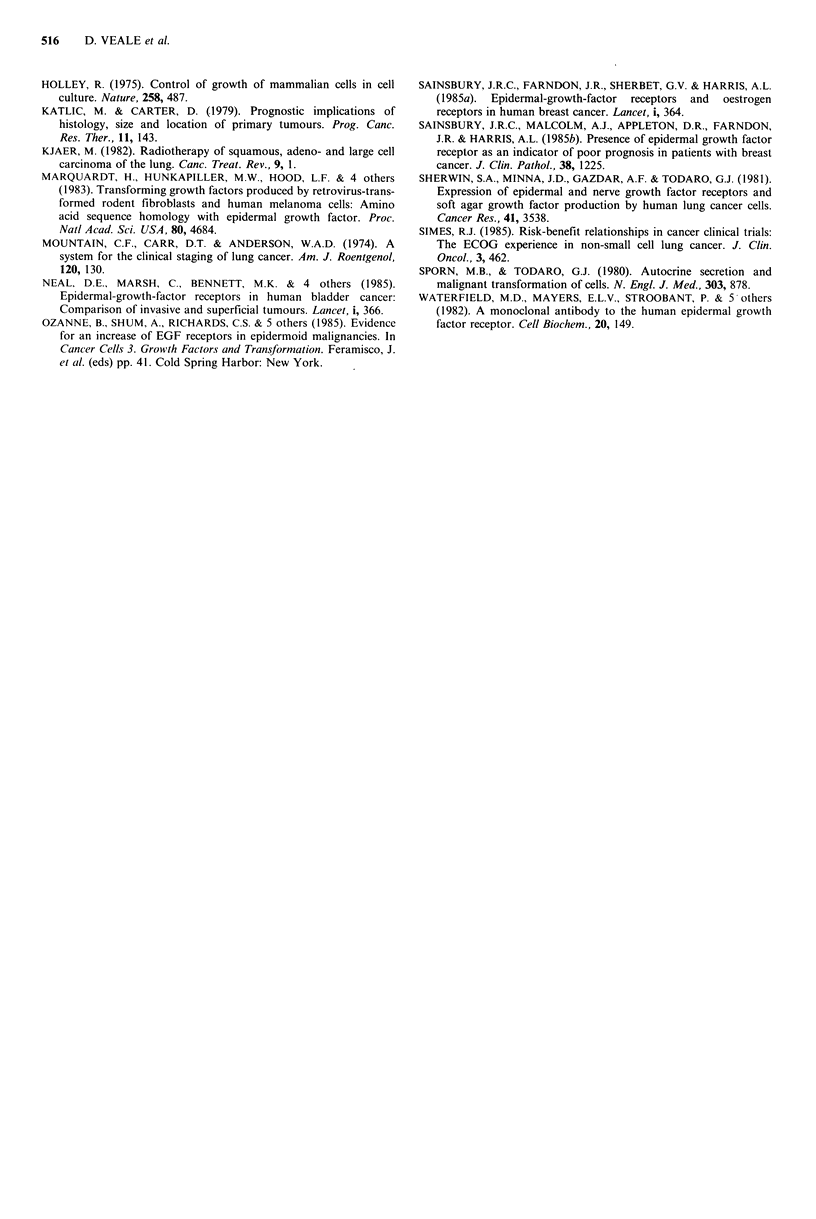# Epidermal growth factor receptors in non-small cell lung cancer.

**DOI:** 10.1038/bjc.1987.104

**Published:** 1987-05

**Authors:** D. Veale, T. Ashcroft, C. Marsh, G. J. Gibson, A. L. Harris

## Abstract

**Images:**


Br. J. Cancer (1987), 55, 513–516 © The Macmillan Press Ltd., 1987

### Epidermal growth factor receptors in non-small cell lung cancer

D. Veale[1], T. Ashcroft[2], C. Marsh[2], G.J. Gibson[1] & A.L. Harris[3]

*Departments of [1]Respiratory Medicine and [2]Pathology, Freeman Hospital; Cancer Research Unit and [3]Department of Clinical Oncology, Newcastle upon Tyne, UK.*

**Summary** The epidermal growth factor receptor is homologous to the oncogene *erb-β* and is the receptor for a class of tumour growth factors (TGF-α). The clinical correlations with its expression were studied in 77 non-small cell lung cancers (NSCLC). They were stained for epidermal growth factor receptor (EGFr) by means of an indirect immunoperoxidase technique using a monoclonal antibody against the receptor. Normal lung tissue and normal bronchus were stained for comparison. Cancer tissue showed significantly increased staining compared to normal lung ($P<0.05$).

Staining for EGFr in 40 squamous carcinomas was significantly stronger than in 37 specimens of other types of NSCLC ($P<0.05$), and staining in stage three NSCLC was stronger than in stage 1 and 2 ($P<0.05$).

These results suggest that the presence of a high intensity of staining for EGF receptor is associated with spread of human non-small cell lung cancer and this receptor may be a suitable target for therapy.

Non-small cell lung cancer (NSCLC) comprises predominantly squamous, adenocarcinomas and large cell undifferentiated tumours. Only 5% of such tumours are curable by surgery (Crofton & Douglas, 1981), and up to 60% are inoperable at the time of diagnosis (Crofton & Douglas, 1981). Chemotherapy is relatively ineffective, with a 17% response rate and no long-term survival (Simes, 1985). Radiotherapy also is rarely curative (Kjaer, 1982). Survival in NSCLC is related to the degree of spread of the tumour at diagnosis, staged by the tumour nodal involvement metastasis (TNM) system (Mountain *et al.*, 1974).

A characteristic of malignant cells in culture is a decreased requirement for serum or supplemental growth factors (Holley, 1975). This suggests that malignant cells may secrete growth factors into their medium. Sporn and Todaro (1980) have suggested the term autocrine growth regulation to describe the situation where a cell secretes a factor, possesses specific receptors for the factor and responds to that factor.

Epidermal growth factor (EGF) is a growth promoting agent found in normal human plasma and tissues which acts by binding to receptors for the hormone on cell surfaces. When injected into newborn mice, EGF elicits the proliferation and differentiation of the epidermis (Cohen, 1983). In tissue culture, EGF induces the proliferation of a wide variety of cell types, including keratinocytes and transformed epithelial cells (Carpenter & Cohen, 1979).

EGF receptor (EGFr) has three parts, an external domain, a transmembrane portion and an internal domain. On binding with its receptor, EGF leads to activation of a tyrosine kinase on the internal domain of the receptor (Carpenter & Cohen, 1979), a property shared with the protein product of some oncogenes. Indeed the *erb*-B oncogene product is a truncated EGFr (Downward *et al.*, 1984). The receptor is also internalised after stimulation.

Peptide growth factors produced by transformed cells include transforming growth factor α (TGF-α), which is structurally related to EGF and binds to the EGF receptor (EGFr) (Marquardt *et al.*, 1983) and transforming growth factor β (TGFβ) which binds to different receptors but is synergistic with TGFα (Marquardt *et al.*, 1983).

In breast cancer, it has been shown that the level of EGFr is associated with metastatic potential, with poorly differentiated tumours and with a poor prognostic subgroup (Sainsbury *et al.*, 1985*a*). The level of EGFr is also associated with the degree of invasion and poor differentiation of bladder cancer (Neal *et al.*, 1985). EGFr have been found on NSCLC cell lines grown in cell culture (Sherwin *et al.*, 1981).

Correspondence: A.L. Harris.
Received 25 July 1986; and in revised form, 5 January 1987.

We have therefore examined lung tumours for the presence of EGFr and investigated the relationship between receptor density, histological subtype and tumour staging. We have examined the relationship of the presence of EGFr on these tumous with prognostic variables such as the degree of differentiation of the tumours.

We used a monoclonal antibody to the receptor in an immunoperoxidase technique (DeLellis *et al.*, 1979).

### Patients and methods

We studied tumour material from 77 patients (53 male and 24 female) undergoing surgery or diagnostic bronchoscopy. The mean age of the patients was 60.4 years. Tumours were staged by the tumour nodal involvement metastasis (TNM) system on examination of the resected material. Tumours with distant metastases or mediastinal nodes are graded stage three, as also are tumours extending into the parietal pleura or involving a main bronchus less than two centimetres from the carina, or any tumour associated with atalectasis or obstructive pneumonitis of an entire lung or pleural effusion,

Forty patients had squamous tumours, of whom 17 had stage three tumours. Twenty patients had adenocarcinomas (six stage three), 13 had large cell undifferentiated carcinomas (five stage three) and four tumours had features of both adeno and squamous carcinomas, two of which were stage three.

Lung tumour samples from operation were frozen in liquid nitrogen in 71 of the patients within 30 minutes of resection. Blocks of tissue were taken from different areas of the tumour and 13 dual samples tested for the presence of EGFr. Six tumour specimens were similarly obtained at bronchoscopy.

The presence of EGFr was also assessed in samples of uninvolved lung in 17 cases and in one sample from a patient without malignancy. Samples of normal bronchus were tested for EGFr in 8 patients.

The EGF receptor was identified by means of an indirect immunoperoxidase technique (DeLellis *et al.*, 1979) with a murine monoclonal antibody (EGFR1) (gift of Dr M. Waterfield). The antibody was raised from an epidermoid carcinoma cell line (A431) which expresses a high concentration of EGF receptors (Waterfield *et al.*, 1982).

Five μm cryostat sections were cut and picked up on lysine-coated slides. After drying with a fan for 30 min, they were fixed in acid for 20 min and washed twice for 5 min in saline buffered to pH 7.6 with *tris*-HCl. The sections were then covered with normal serum (diluted 1:4 with *tris*-buffered saline) as a blocking agent for 10 min. The sections were incubated at room temperature with primary antibody for 30 min. After two washes in *tris*-buffered saline, the blocking step was repeated. The sections were then incubated with peroxidase-conjugated rabbit anti-mouse immunoglobulin (Dakopatts) for 30 min. After two further 5-min washes, peroxidase activity was developed by means of a solution of diaminobenzidine. The sections were washed in water, counterstained with haemaoxylin, dehydrated and mounted. The positive control was human placenta, which contains large numbers of EGFr. For negative controls we omitted the primary antibody. Controls used in each run showed similar intensity of staining.

The intensity of staining was assessed by two observers reading the sections independently without knowledge of the tumour stage or degree of differentiation. The sections were graded on a scale from 0 to + + + according to the intensity of staining of malignant cells relative to the positive control. The placental control stained strongly positively on all occasions and showed no staining when the primary antibody was omitted. The histological typing of the tumours was carried out on paraffin sections of the resected material. Statistical analysis was performed using the chi-square test on a two by four table of results.

### Results

*Normal lung and bronchus EGFr*

Results of staining for EGFr in 17 specimens of normal lung showed no staining in 5 specimens and mild staining (1+) in 9. Only two specimens of normal lung tissue showed moderate staining graded + +, while one specimen had strong staining for EGFr. In normal bronchial epithelium there was strong positive staining for EGFr in a thin band in the basal layer (Figure 1) and weaker staining in bronchial glands.

**Figure 1** Immunoperoxidase staining for EGFr in normal bronchial tissue showing strong positive staining in the basal layer. (×240).

*Carcinoma EGFr – Relation to histological type and stage*

Staining in cancer specimens was significantly stronger in tumour than in normal lung tissue ($\chi^2 = 8.47$, $P < 0.01$). The results for staining for EGFr in tumours are snown in Table I, and illustrated in Figure 2. Staining in sections from different parts of the tumour showed a good correlation of intensity of EGFr staining in 10 cancers examined. Eight of 10 tumours examined had identical grading from different areas. No tumour graded EGFr negative was then graded positive but one tumour which graded positive was subsequently negative.

Twenty-seven of forty squamous tumours stained moderately strongly or strongly (+ + or + + +), while only nine of twenty adenocarcinomas stained similarly. Overall, the staining in squamous tumours was significantly stronger than for other NSCLC ($\chi^2 = 8.88$, $P < 0.05$). When all the tumours were grouped according to stage there was significantly stronger staining in the 30 stage three tumours compared to 47 stage one and stage two tumours ($\chi^2 = 9.87$, $P < 0.05$) (Table II). There was no significant difference in the number of stage three squamous tumours (17/40) compared to the number of stage three adeno and large cell carcinomas (13/37) ($\chi^2 = 0.44$) (Table III).

When all the tumours were divided according to degree of differentiation (well and moderately differentiated versus

**Figure 2** Immunoperoxidase staining for EGFr in a squamous lung cancer. (×57). (a) primary antibody omitted; (b) monoclonal primary antibody.

**Table I** EGFr in non-small cell lung cancer (normal lung and bronchus)

| | Staining grade | | | | |
|---|---|---|---|---|---|
| | *0* | *1* | *2* | *3* | *Total* |
| *NSCLC* | | | | | |
| Squamous | 5 | 8 | 13 | 14 | 40 |
| Adeno | 6 | 5 | 8 | 1 | 20 |
| Large cell | 4 | 3 | 5 | 1 | 13 |
| Adenosquamous | 0 | 1 | 2 | 1 | 4 |
| Lung | 5 | 9 | 2 | 1 | 17 |
| Bronchus | 2 | 5 | 0 | 0 | 7 |

**Table II** EGFr staining re tumour stage

| | Staining grade | | | | |
|---|---|---|---|---|---|
| | *0* | *1* | *2* | *3* | *Total* |
| Stages 1 and 2 | 14 | 7 | 17 | 9 | 47 |
| Stage 3 | 1 | 10 | 11 | 8 | 30 |

**Table III** Relation of stage to histological subtype

| | *Squamous* | *Non-squamous* |
|---|---|---|
| Stages I and II | 23 | 24 |
| Stage III | 17 | 13 |
| | 40 | 37 |

NS ($\chi^2 = 0.44$).

poorly differentiated), there was no significant difference in intensity of staining for EGFr between the groups. Likewise squamous tumours alone showed no significant relationship between EGFr score and degree of differentiation, although there were only two well differentiated squamous tumours. There was no statistically significant difference in the degree of differentiation in different stages of tumour spread.

### Discussion

We have demonstrated the presence of epidermal growth factor receptors in non-small cell lung cancer and the fact that squamous carcinomas show more staining than non-squamous tumours. We have also shown that the intensity of staining for EGFr is related to the stage of spread of the tumours.

The histology of NSCLC can vary throughout the tumour (Mountain *et al.*, 1974) and thus we have taken samples from different areas of the tumours and found no significant variation of staining within tumours.

Hendler and Ozanne (1984) demonstrated EGFr in 11 squamous lung cancers. They showed that 7 of 8 adenocarcinomas failed to stain for EGFr, using a semiquantitative autoradiography technique. The greater intensity of staining for EGFr in squamous tumours is consistent with the findings that squamous tumours at other sites, such as head and neck or cervical cancer, and squamous carcinoma cell lines express more EGF receptors than other cancers (Cowley *et al.*, 1984). Ozanne *et al.* have shown a 2 to 10-fold excess of receptor sites in squamous cancers of various sites, including lung, compared to normal skin (Gusterson *et al.*, 1984).

In our series, normal lung showed far less staining for EGFr, and only 1/17 normal lungs were strongly positive. This may reflect individual variation or possibly EGFr involvement in other chronic inflammatory processes.

Our results suggest that epidermal growth factor receptor may play a part in the genesis or spread of lung cancers. This is the third common primary carcinoma in which we have found a correlation of the presence of EGFr with some of the prognostic features (Neal *et al.*, 1985; Sainsbury *et al.*, 1985*b*). This may suggest that, rather than EGFr being involved in tumour initiation, subclones of tumours that express more EGFr or have had rearrangement of EGFr genomic DNA may be selected for growth, invasion and metastasis. This may be a relatively late change in tumour development.

Cerny *et al.* (1986) examined 63 lung tumours, mainly bronchial biopsies, and found 86% of squamous cancers stained positively for EGFr, but none of 15 small cell tumours stained. However, only 5 adenocarcinomas or large cell tumours were examined and the relation to stage was not assessed.

Neal *et al* (1985) showed that staining for EGFr was related to poor differentiation of bladder cancers, and Sainsbury *et al.* (1985*b*) found a similar relationship in breast cancers, but we did not observe this in our specimens. In our series of bronchial tumours, there was no significant association of differentiation with stage and this may be a reflection of the large proportion of tumours that were moderately or poorly differentiated. Indeed in adenocarcinoma of the bronchus there is no significant survival difference as regards degree of differentiation (Katlic & Carter, 1979).

The detection of normal basal cells in the bronchial mucosa staining for EGFr, first reported by Gusterson *et al.* (1984), suggests that these stem cells of the mucosa may require EGF for proliferation and differentiation to normal mucosal cells. If these cells are the site of transformation for lung cancers, it would be expected that some adenocarcinomas and large cell tumours would also express EGFr, as we have found, in contrast to Ozanne's group (Hendler & Ozanne, 1984).

The demonstration of a high concentration of EGFr in the worse prognosis NSCLC suggests a novel potential target for therapy. To quantitate the binding characteristics, we have assayed membranes from these primary tumours and have found high affinity sites (Kd $1 \times 10^{-9}$ molar) for EGF and much higher numbers of receptors than breast cancers (190 fmol mg$^{-1}$ membrane protein versus 4–47 fmol mg$^{-1}$ membrane protein) (Sainsbury *et al.*, 1985*a*). EGF bound drug would be expected to react specifically at these sites, thus leading to higher concentration of drug in tumour than in other tissue. Drugs linked to EGF should be more selective than current therapies, particularly as receptor stimulation is linked to internalisation, and squamous tumours express several-fold more EGFr than reported for other normal tissues, including normal keratinocytes (Ozanne *et al.*, 1985).

The monoclonal antibody used here recognises the external domain of the EGFr, so this could also be used for targeting. The relationship of EGFr to other prognostic features in three common primary tumours shows that this type of drug therapy might be generally applicable in patients in whom there is little alternative therapy at present. We are currently studying this approach in human non-small cell cancer lines.

We thank Mr G.N. Morritt, Mr C. Hilton, Mr A. Hedley-Brown and Mr A. Blesovsky for help in collection of tumour specimens and Dr S. Nariman for permission to collect bronchoscopy specimens. We thank Dr M. Waterfield for kindly donating the antibodies to EGF receptor.

### References

CARPENTER, G. & COHEN, S. (1979). Epidermal growth factor. *Ann. Rev. Biochem.*, **48,** 193.

CERNY, T., BARNES, D.M., HASLETON, P.S. & 4 others (1986). Expression of epidermal growth factor receptor (EGF-R) in human lung tumours. *Br. J. Cancer,* **54,** 265.

COHEN, S. (1983). The epidermal growth factor. *Cancer,* **51,** 1787.

COWLEY, G., SMITH, J., GUSTERSON, B., HENDLER, F. & OZANNE, B. (1984). The amount of EGF receptor is elevated on squamous cell carcinomas. In *The Cancer Cell,* p. 5. Cold Spring Harbor: New York.

CROFTON, J. & DOUGLAS, A. (1981). In *Respiratory Diseases,* 3rd ed., p. 653. Blackwell.

DELELLIS, R.A., STERNBERGER, L.A., MANN, R.B., BANKS, P.M. & NAKANE, P.K. (1979). Immunoperoxidase technics in diagnostic pathology. *Am. J. Clin. Path.*, **71,** 483.

DOWNWARD, J., YARDEN, Y., MAYER, L. & 6 others (1984). Close similarity of epidermal growth factor receptor and v-erb-b oncogene protein sequences. *Nature,* **307,** 521.

GUSTERSON, M., COWLEY, G., SMITH, J.A. & OZANNE, A. (1984). Cellular localisation of human epidermal growth factor receptor. *Cell Biol. Internat. Rep.*, **8,** 649.

HENDLER, F.J. & OZANNE, B.W. (1984). Human squamous cell lung cancers express increased epidermal growth factor receptors. *J. Clin. Invest.*, **74,** 647.

HOLLEY, R. (1975). Control of growth of mammalian cells in cell culture. *Nature*, **258**, 487.

KATLIC, M. & CARTER, D. (1979). Prognostic implications of histology, size and location of primary tumours. *Prog. Canc. Res. Ther.*, **11**, 143.

KJAER, M. (1982). Radiotherapy of squamous, adeno- and large cell carcinoma of the lung. *Canc. Treat. Rev.*, **9**, 1.

MARQUARDT, H., HUNKAPILLER, M.W., HOOD, L.F. & 4 others (1983). Transforming growth factors produced by retrovirus-transformed rodent fibroblasts and human melanoma cells: Amino acid sequence homology with epidermal growth factor. *Proc. Natl Acad. Sci. USA*, **80**, 4684.

MOUNTAIN, C.F., CARR, D.T. & ANDERSON, W.A.D. (1974). A system for the clinical staging of lung cancer. *Am. J. Roentgenol*, **120**, 130.

NEAL, D.E., MARSH, C., BENNETT, M.K. & 4 others (1985). Epidermal-growth-factor receptors in human bladder cancer: Comparison of invasive and superficial tumours. *Lancet*, **i**, 366.

OZANNE, B., SHUM, A., RICHARDS, C.S. & 5 others (1985). Evidence for an increase of EGF receptors in epidermoid malignancies. In *Cancer Cells 3. Growth Factors and Transformation*. Feramisco, J. *et al.* (eds) pp. 41. Cold Spring Harbor: New York.

SAINSBURY, J.R.C., FARNDON, J.R., SHERBET, G.V. & HARRIS, A.L. (1985*a*). Epidermal-growth-factor receptors and oestrogen receptors in human breast cancer. *Lancet*, **i**, 364.

SAINSBURY, J.R.C., MALCOLM, A.J., APPLETON, D.R., FARNDON, J.R. & HARRIS, A.L. (1985*b*). Presence of epidermal growth factor receptor as an indicator of poor prognosis in patients with breast cancer. *J. Clin. Pathol.*, **38**, 1225.

SHERWIN, S.A., MINNA, J.D., GAZDAR, A.F. & TODARO, G.J. (1981). Expression of epidermal and nerve growth factor receptors and soft agar growth factor production by human lung cancer cells. *Cancer Res.*, **41**, 3538.

SIMES, R.J. (1985). Risk-benefit relationships in cancer clinical trials: The ECOG experience in non-small cell lung cancer. *J. Clin. Oncol.*, **3**, 462.

SPORN, M.B., & TODARO, G.J. (1980). Autocrine secretion and malignant transformation of cells. *N. Engl. J. Med.*, **303**, 878.

WATERFIELD, M.D., MAYERS, E.L.V., STROOBANT, P. & 5 others (1982). A monoclonal antibody to the human epidermal growth factor receptor. *Cell Biochem.*, **20**, 149.